# Fabricating Oral Disintegrating Tablets Without Disintegrant Using Powder-Based 3D Printing

**DOI:** 10.3390/pharmaceutics17040435

**Published:** 2025-03-28

**Authors:** Jiu Wang, Shunfang Liu, Minmei Lin, Peihong Chen, Huagui Yi, Zhufen Lv, Yuanfen Liu

**Affiliations:** 1 Guangdong Provincial Key Laboratory for Research and Evaluation of Pharmaceutical Preparations, Center for New Drug Research and Development, Guangdong Pharmaceutical University, Guangzhou 510006, China; wangjiu@gdpu.edu.cn (J.W.); gdpulmm@163.com (M.L.); yhg20020125@163.com (H.Y.); 2 Department of Pharmacy, Jiangsu Health Vocational College, Nanjing 211800, China; 3 Guangdong High Education Institutes Engineering Research Center of Modified-Released Pharmaceutical Products, School of Traditional Chinese Medicine, Guangdong Pharmaceutical University, Guangzhou 510006, China; 4 Guangdong Provincial Engineering Center of Topical Precision Drug Delivery System, School of Pharmacy, Guangdong Pharmaceutical University, Guangzhou 510006, China; 5 Jieyang People’s Hospital, Jieyang 522000, China

**Keywords:** powder-based 3D printing, orally disintegrating tablets, central composite design

## Abstract

**Background**: Powder-based 3D printing, an advanced additive manufacturing technique, can produce oral disintegrating tablets (ODTs) without disintegrants, creating larger-pored tablets via layer-by-layer powder stacking for better water absorption than traditional tablets. **Methods**: This study focused on using powder-based 3D printing to fabricate clozapine-based ODTs. Through central composite design (CCD), the formulation of ODTs was optimized for rapid disintegration. Analytical techniques such as X-ray Powder Diffraction (XRD), Fourier Transform Infrared Spectroscopy (FTIR), and Differential Scanning Calorimetry (DSC) were employed to investigate the compatibility between clozapine and excipients. **Results**: The optimized 3D-printed ODTs exhibited a remarkably short disintegration time of (9.9 ± 0.7) s compared to (40) s for compressed tablets. The contact angle of the 3D-printed ODTs was measured as 60.48 ± 0.36°, indicating favorable wettability for disintegration. Scanning Electron Microscopy (SEM) analysis revealed a porous structure in 3D-printed tablets, with a porosity of 48.97% (over two times higher than that of compressed tablets as determined by mercury injection meter). **Conclusions**: Collectively, this finding demonstrates the feasibility of fabricating highly hydrophilic and non-distensible ODTs without disintegrants using powder-based 3D printing.

## 1. Introduction

Oral administration remains the preferred drug delivery method due to its simplicity, safety, and patient compliance [1]. However, traditional solid dosage forms pose challenges for populations with dysphagia, such as the elderly and children, necessitating specialized solutions like oral disintegrating tablets [2,3,4]. ODTs rapidly disintegrate in the oral cavity without water, enhancing medication adherence for patients with swallowing difficulties or requiring urgent treatment [5,6,7]. Their porous structure and hydrophilic excipients enable fast wetting and drug dissolution [8,9]. Conventional ODT manufacturing methods, such as direct compression, often compromise porosity for mechanical strength, leading to slower disintegration and reliance on moisture-sensitive disintegrants [10,11,12].

Powder-based 3D printing has emerged as a transformative approach, enabling precise control over tablet architecture and drug release profiles. Technologies like binder jetting facilitate mass production of rapid-release formulations (e.g., Spritam) while optimizing porosity and dissolution kinetics [13,14,15,16,17,18]. Despite these advances, research on 3D-printed ODTs (3DP ODTs) remains limited, particularly regarding formulation optimization, porosity effects, friability, and storage stability [19,20,21].

Clozapine, a critical antipsychotic for treatment-resistant schizophrenia, faces compliance challenges due to patient refusal or covert non-adherence [22,23]. Existing clozapine ODTs disintegrate within 60 s, but faster disintegration could improve administration success in uncooperative patients [24,25]. Recent studies suggest 3D printing can achieve rapid disintegration through pore structure modulation, eliminating conventional disintegrants and moisture sensitivity issues [26].

This study aims to address these gaps by investigating the application of Powder Bed 3D Printing to fabricate clozapine ODTs without disintegrants. By optimizing the formulation and employing various characterization techniques, we seek to develop a more efficient and stable method for ODT preparation, especially benefiting patients who require rapid drug absorption and face challenges with traditional tablet administration. Additionally, we aim to compare the 3D printing process with traditional methods through various analyses and elucidate the underlying mechanisms of observed differences, thereby providing valuable insights for the development of more efficient and stable ODTs.

## 2. Materials and Methods

### 2.1. Materials

Clozapine was obtained from Prosperity Galaxy Chemical Co., Ltd. (Wuhan, Hubei, China). Clozapine oral disintegrating tablets (25 mg, directly compressed) were obtained from Nhwa Pharma. Co., Ltd. (Xuzhou, Jiangsu, China). The excipients used in 3D ODTs were as follows: mannitol (Roquette, Lestrem, France), sucrose (Nanning Pharmaceutical Co., Ltd., Nanning, Guangxi, China), microcrystalline cellulose (MCC, Shenmei Pharmaceutical Technology Co., Ltd., Shanghai, China), polyvinylpyrrolidone K30 (PVP K30, Zhanwang Pharmaceutical Co., Ltd., Jiaxing, Zhejiang, China) and crospovidone (Sunhere Pharmaceutical Excipients Co., Ltd., Huainan, Anhui, China). Other reagents mentioned in the manuscript were chromato-graphical or analytical grade. Powder-based 3D printing machine (LTY-200, Folichif, Shanghai, China). Hardness tester (YPD-200C, Huanghai Medicine Drug Testing Instruments Co., Ltd., Shanghai, China). High-performance liquid chromatography (HPLC) (Agilent 1260, Agilent, Santa Clara, CA, USA). C-18 column (4.6 mm × 250 mm, YMC-Triart, Kyoto, Japan). Contact Angle Meter SDC-200 (Shending Scientific Instrument, Dongguan, Guangdong, China). A texture analyzer (TA.XT Plus, Stable Micro System, Godalming, UK). Differential scanning calorimetry (DSC), (DSC4000 PerkinElmer, Waltham, MA, USA). Fourier transform infrared spectrophotometer (FTIR), (Bruker Alpha-T, Ettlingen, Germany). X-ray powder diffractometer (XRD), (Bruker D8, Bruker, Karlsruhe, Germany). Scanning electron microscopy (SEM), (Helois Nanolab 600i, FEI NanoPorts, Hillsboro, OR, USA). UV-Vis spectrophotometer (UV-2700, Shimadzu Corporation, Kyoto, Japan).

### 2.2. Method of Preparation of 3DP Clozapine ODTs

**Figure 1:**
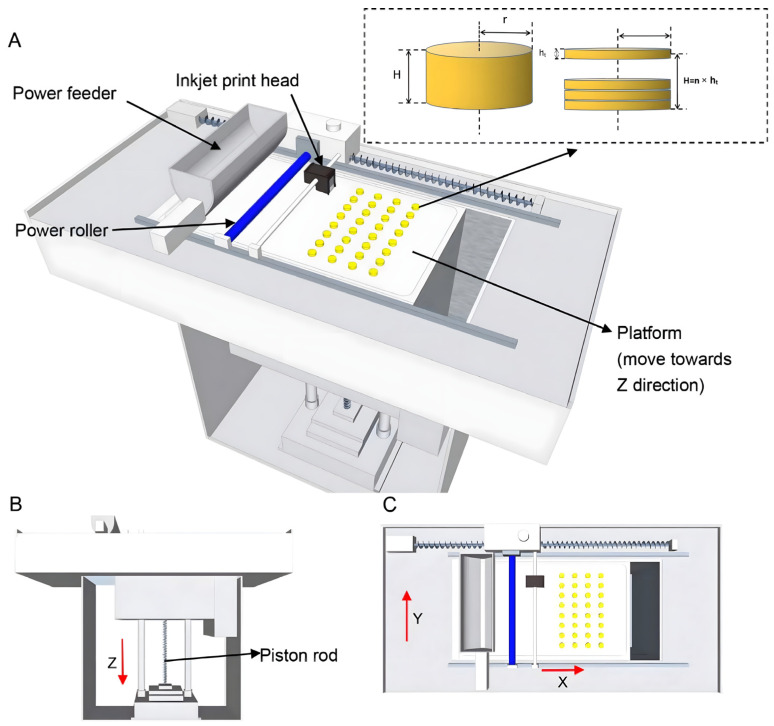
(**A**) Full view of the powder-based 3D printing machine. (**B**) Front view of the powder-based 3D printing machine. (**C**) Top view of the powder-based 3D printing machine.

A three-dimensional geometric design for the tablets, along with the relevant parameters, was programmed using SolidWorks 2017 software (Version 24.0 SP5, Dassault Systèmes). Subsequently, the design was exported as a file with a.stl extension. The powder-based 3D printing machine (Figure 1) was employed for the fabrication process. Firstly, the machine automatically smoothed the mixed powder, which was supplied by the powder feeder, using a powder roller across the surface of the working platform. Subsequently, the binding liquid (a mixture of ethanol and water in a certain ratio) within the print head was selectively sprayed onto the powder in the X-Y orientation, following the printing parameters, to form the predetermined pattern. Upon completion of printing a single layer, the working platform was lowered along the *Z*-axis to a preset layer thickness by means of the piston rod. A fresh layer of powder was then spread over the surface, and the aforementioned steps were repeated until the 3D shape programmed within the software was fully fabricated. Notably, all the powders used were sieved through a mesh with a pore size of 125 μm, and each batch comprised the production of 50 tablets. The proportion of the components in the prescription for the 3D powder-based clozapine ODTs is presented in Table 1.

### 2.3. The Formulation Optimization of 3D Powder-Based Clozapine ODTs

#### 2.3.1. Single-Factor Experiments

Following the results of preliminary formulation screening experiments, it was established that the ratio of mannitol to sucrose, the ratio of MCC to PVP K30, and the ethanol concentration were the key compositional factors influencing the hardness and disintegration time of the powder-based 3DP ODTs.

#### 2.3.2. Central Composite Design (CCD)

A central composite design was employed to optimize the formulation of these 3D powder-based ODTs. The concentration of ethanol (X_1_), the mannitol–sucrose ratio (X_2_) and the MCC : PVP K30 ratio (X_3_) were selected as independent variables. Meanwhile, the hardness (*Y*_1_) and disintegration time (*Y*_2_) of the tablets were chosen as the dependent variables. A 3-factorial level central composite design (CCD) was implemented and subjected to statistical analysis using Design Expert 8.0.6 software. In this design, the concentration of ethanol (X_1_), the mannitol-to-sucrose ratio (X_2_), and the MCC-to-PVP K30 ratio (X_3_) were designated as independent variables, while the hardness (*Y*_1_) and disintegration time (*Y*_2_) of the tablets were identified as dependent variables. A 3-factorial level CCD was conducted and analyzed statistically using Design Expert 8.0.6 software.

### 2.4. Characterization of ODTs

#### 2.4.1. Hardness

The hardness of the tablets was measured using a hardness tester. Six tablets were selected for each measurement.

#### 2.4.2. Determination of Drug Content

In alignment with the analytical methodologies prescribed by the United States Pharmacopeia and the guidelines established by the U.S. Food and Drug Administration (FDA), ten clozapine ODTs were selected from each batch for content uniformity testing. Whenever feasible, the content of each tablet was measured individually, adhering to the principle of harmonization with international standards. Our methodology aligns with these guidelines and has been slightly modified to meet the specific requirements of the Chinese Pharmacopoeia (ChP.) 2020 Edition (Volume II), which serves as a reference for our study. The quantification of clozapine was conducted using HPLC equipped with a C-18 column. The mobile phase, flowing at a rate of 1 mL/min, was composed of methanol: 0.4% triethylamine aqueous solution (70:30, *v*/*v*). The injection volume was set at 20 μL, and the UV absorbance was recorded at a wavelength of 257 nm.

#### 2.4.3. Contact Angle Testing

The surface contact angle of clozapine ODTs was measured utilizing a contact angle, equipped with an automated high-speed imaging module. A microsyringe needle was used to deposit a 3 μL droplet of deionized distilled water onto the surface of the tablet. Images were captured automatically within 1 s to reduce hydration effects. Three measurements were taken on three tablets, and results are given as mean ± SD.

#### 2.4.4. In Vitro Disintegration by Texture Analyzer

A texture analyzer was utilized to evaluate the disintegration process and determine the disintegration time of two types of ODTs, with dissolution media composed of distilled water (pH 6.8, 37 °C) to simulate oral conditions [27]. The tablets were affixed using double-sided adhesive to ensure secure attachment to the probe, thereby preserving the structural integrity of the tablets. The texture analyzer moved towards a vessel with 4 mL of distilled water at 5 mm/s after attaching the tablet to the probe. Upon immersion in the medium and contact with a submerged perforated platform, a trigger force of 3.0 g was applied. Subsequently, the texture analyzer increased and sustained the force at 10.0 g, which was deemed sufficient to measure the complete disintegration of the sample, for a specified duration. Throughout the disintegration process, the texture analyzer maintained this force while recording the penetration distance. The distance–time curves generated by the texture analyzer software (Exponent (Version 6.2), Stable Micro System, Godalming, UK) enabled the calculation of disintegration time and facilitated the analysis of disintegration behavior. A total of six tablets were randomly selected from each type of ODT for measurement.

#### 2.4.5. In Vitro Dissolution Test

Six tablets were randomly selected to determine the dissolution of 3DP ODTs and direct-pressed ODTs. Using the dissolution test method (ChP. 2020, Part IV, General Chapter 0931, Basket Method), hydrochloric acid solution was the dissolution medium, with a rotation speed of 100 rpm. At 0.5, 1, 2, 3, 4, and 5 min, about 10 mL of solution was taken and replaced with the same volume of dissolution medium. The collected solution was filtered, and its absorbance was measured at 240 nm via UV-Vis spectrophotometry.

#### 2.4.6. Porosity Measurement Method

Two clozapine tablets, manufactured via distinct processes, were randomly selected for analysis using a mercury injection meter. Initially, the samples were placed in an oven for two hours to eliminate moisture content. Following this, the samples were weighed and loaded into the inflator, which was subsequently sealed and weighed again. The inflator was then positioned in the low-pressure station for further analysis.

#### 2.4.7. Differential Scanning Calorimetry

The thermal properties of clozapine, its physical mixture, and the clozapine 3DP ODTs were analyzed using DSC. Approximately 5 mg of each sample was precisely weighed and placed in aluminum pans. Prior to analysis, the samples were stabilized by maintaining them at 30 °C for 10 min. The samples were then subjected to a temperature increase from 30 to 300 °C at a rate of 10 °C/min under a nitrogen flow of 20 mL/min.

#### 2.4.8. Fourier Transform Infrared Spectroscopy

FTIR spectra of clozapine, the physical mixture, and the clozapine 3DP ODTs were obtained using FTIR with potassium bromide (KBr) pellets. Specifically, 2 mg of the sample was mixed with 100 mg of dried KBr, ground together, and compressed into a uniform, smooth, and transparent pellet. The samples were scanned three times over the wavelength range from 4000 to 400 cm^−1^.

#### 2.4.9. X-Ray Powder Diffraction

X-ray diffractograms of clozapine, its physical mixture, and the clozapine 3DP ODTs were generated using an XRD. The analysis was carried out using a copper target, with the tube pressure and current set to 40 kV and 40 mA, respectively. Diffraction peaks were scanned over a range of 5° and 50° at 2θ with a scanning speed of 6°/min.

#### 2.4.10. Scanning Electron Microscopy

The micromorphology of the clozapine 3DP ODTs and direct-pressed ODTs was examined using SEM. Samples were affixed to an aluminum disc using carbon tape, and an ion-sputtering device (150T, EMS, Lewes, East Sussex, UK) was employed to coat the sample with gold powder prior to observation.

#### 2.4.11. Disintegration Process

The 3DP ODTs and direct-pressed ODTs were randomly selected, with one tablet taken from each type. These tablets were simultaneously placed into a container filled with dissolution media maintained at a constant temperature, and the disintegration process of both tablet types was observed. This procedure was repeated three times.

## 3. Results and Discussion

### 3.1. The Result of Formulation Optimization for 3D Powder-Based Clozapine ODTs

**Figure 2:**
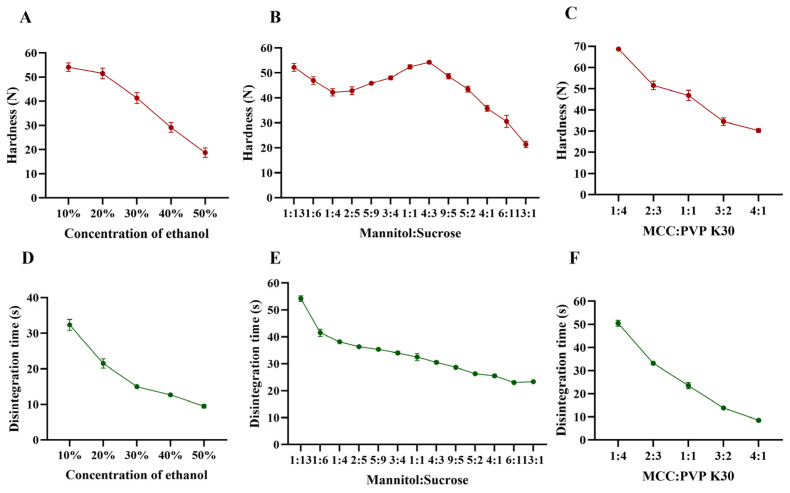
Effect of formulation on hardness and disintegration time. (**A**,**D**) Concentration of ethanol, (**B**,**E**) mannitol–sucrose, (**C**,**F**) MCC : PVP K30.

The disintegration rate and hardness of the tablets were influenced by multiple factors. As shown in Figure 2A–F, an increase in ethanol content, changes in the mannitol–sucrose ratio, and variations in the MCC : PVP K30 ratio affected these parameters. Specifically, as the ethanol content and MCC : PVP K30 ratio increased, the hardness decreased (Figure 2A,C), while the disintegration rate of the tablets accelerated (Figure 2D,F). This can be attributed to the reduction in water content, which acts as a solvent in the bonding process, and the decrease in the binder PVP K30. The interaction between MCC and PVP K30 was also evaluated in terms of disintegration time and tablet hardness. PVP K30 exhibited cohesive properties, enhancing the internal cohesive force of the tablet to improve its strength. Although PVP K30 facilitates the binding of tablet particles, it does not hinder water penetration due to its wicking properties, which instead aid in disrupting hydrogen bonds between particles and expedite tablet disaggregation [28]. MCC, a highly effective wicking agent, enhances rapid water penetration into the tablet matrix, thereby accelerating disintegration [29]. Given that MCC is insoluble in both water and ethanol, an increase in the MCC-to-PVP K30 ratio results in a reduction in both tablet hardness and disintegration time.

The effects of mannitol and direct-pressed ODT on hardness and disintegration rate are displayed in Figure 2. The disintegration rate of the tablets accelerated as the mannitol–sucrose ratio increased. Figure 2B demonstrated the influence on hardness when the mannitol–sucrose ratio was varied from 1:13 to 13:1. A peak shape appeared within the range of 1:4 to 13:1, with the maximum hardness achieved at a 4:3 ratio. Sucrose functions as an adhesive agent. The observed changes in hardness within this ratio range may be attributed to potential synergistic interactions between mannitol and sucrose. During the printing process, sucrose dissolves in water, which can enhance cohesion, causing the mannitol–sucrose mixture to re-solidify as larger particles within the internal tablet matrix, thereby creating gaps between adjacent particles [30]. The small particle size of mannitol enabled it to fill these gaps, enhancing the bonding effect between neighboring particles. There appears to be an optimal ratio at which the remaining mannitol effectively fills the inter-particle gaps in the re-solidified particles, acting as a connector to link adjacent particles and build strong bridges that contribute to the highest hardness [31]. As the mannitol–sucrose ratio continued to increase, the decreased proportion of sucrose could no longer provide sufficient adhesion for the additional mannitol in tablets, resulting in decreased hardness [32]. Liquid penetration was identified as the rate-determining step in disintegration. When sucrose dissolved from the pore walls, it increased the viscosity of the liquid phase [33], thereby slowing water penetration and reducing the disintegration time as the mannitol–sucrose ratio decreased from 1:13 to 13:1.

Single-factor experiment results supported the determination of appropriate ranges for CCD optimization. The optimization aimed to achieve tablets with sufficient hardness (greater than 30 N to achieve adequate strength for transport) and disintegration time (less than 30 s, meeting half of the disintegration time requirement specified in the Chinese Pharmacopeia 2020). Combined analysis led to the determination of appropriate ranges for CCD optimization. The concentration range of ethanol for CCD was set at 10–40%, while the ratios of both mannitol–sucrose and MCC : PVP K30 were defined as 1:1 to 4:1. Further studies on CCD will help clarify the roles of each auxiliary material and the disintegration mechanism of 3DP ODTs.

### 3.2. Formulation Optimization by the CCD

Using Design Expert modeling, quad models were used to assess hardness (*Y*_1_) and disintegration time (*Y*_2_). The model equations based on the coded values for *Y*_1_ and *Y*_2_ are expressed by Equations (1) and (2), respectively.

(1) $$Y_{1}=35.65-4.12-4.63B-3.71C+1.09AB+0.77AC+1.02BC-1.32A^{2}+0.091B^{2}+1.10C^{2}$$

(2) $$Y_{2}=8.83-2.93A-3.51B-1.57C+2.04AB+0.31AC-0.062BC+0.91A^{2}+1.48B^{2}+0.79C^{2}$$

The magnitude and sign of the coefficients in these polynomial equations were used to infer the effects of the respective terms [34]. X_1_, X_2_, and X_3_ had significant negative coefficient values, indicating that an increase in each variable by one unit would reduce the corresponding response values of *Y*_1_ and *Y*_2_.

**Figure 3:**
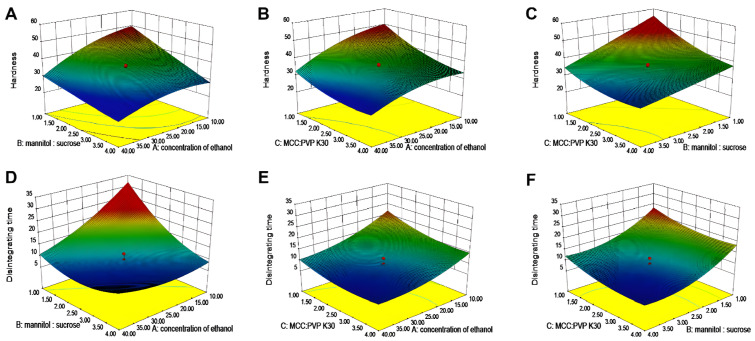
The 3D response surface for prescription screening. (**A**–**C**) Hardness. (**D**–**F**) Disintegration time.

As shown in Figure 3, 3D response surface plots were instrumental in studying the interaction effects of two independent variables on the dependent variables [35]. Figure 3A showed that the 3D surface areas increased with a decrease in ethanol concentration and mannitol–sucrose ratio, suggesting that any reduction in these variables had a positive impact on the surface areas. The X_m_X_n_ term in the polynomial equations also reflects the blending behavior of the independent variables. Equation (1), representing the response surface for *Y*_1_, had positive terms indicating synergistic behavior between X_1_ and X_2_ with a coefficient of +1.09, between X_1_ and X_3_ with a coefficient of +0.77, and between X_2_ and X_3_ with a coefficient of +1.02. In Equation (2), the signs of the coefficients of X_1_X_2_ and X_1_X_3_ are both positive, demonstrating that these components enhanced each other’s effects. However, X_2_X_3_ had a negative coefficient of −0.062, suggesting a slightly antagonistic behavior where the variables counteracted each other’s effects. The results of single-factor and CCD experiments demonstrated the influence of each factor on hardness and disintegration time of tablets. In this study, clozapine ODT formulations were optimized to reduce disintegration time while ensuring adequate tablet hardness. Based on the numerical optimization and the design goal, the desirable range of responses was 35 N < *Y*_1_ < 40 N and 6 s < *Y*_2_ < 10 s. The optimized formulation predicted by Design Expert 8.0.6 was 35% ethanol (X_1_), mannitol–sucrose ratio (X_2_) of 1.90, and MCC : PVP K30 ratio (X_3_) of 1.65. The predicted and experimental values were in strong agreement, with the observed values of *Y*_1_ and *Y*_2_ falling within the 95% prediction interval (*Y*_1_: 32.78–38.52; *Y*_2_: 6.53–11.12), respectively. This confirmed the validity of the generated mathematical equation for predicting *Y*_1_ and *Y*_2_. With this optimized formulation, clozapine ODTs fabricated by 3D powder-based printing disintegrate within 10 s. Utilizing ethanol as a solvent for the binder PVP K30, a reduction in ethanol concentration mitigates excessive particle consolidation. Furthermore, the balance between MCC and PVP K30 not only ensures the hardness of the tablets but also prevents pore blockage due to an overabundance of binder. This optimization strategy illustrates that by regulating multiple variables, it is feasible to concurrently satisfy the requirements for hardness and disintegration performance.

### 3.3. Characterization Study of ODTs

#### 3.3.1. Content Uniformity and Hardness

The 3DP ODTs were prepared according to the CCD optimization results. The contents (mean ± SD) of three batches of clozapine 3DP oral disintegrated tablets were (25.4 ± 0.3) mg, (25.2 ± 0.4) mg and (25.0 ± 0.2) mg, respectively, and the hardness was (35.88 ± 0.43) N, all meeting the requirements of ChP. 2020.

#### 3.3.2. Clozapine and Excipient Interactions

**Figure 4:**
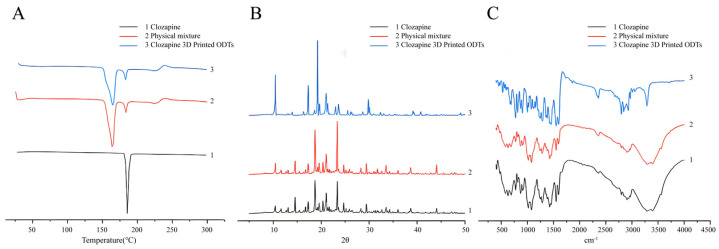
Exploring the compatibility of clozapine and excipients. (**A**) DSC thermograms of clozapine, physical mixture and clozapine 3DP ODTs. (**B**) X-ray powder diffractograms of clozapine, physical mixture and clozapine 3DP ODTs. (**C**) FTIR spectra of clozapine, physical mixture and clozapine 3DP ODTs.

DSC, FTIR and XRD were utilized to investigate potential interactions between the clozapine active pharmaceutical ingredient and excipients in the powder 3D printing process for ODT preparation. DSC thermograms of clozapine, physical mixture and clozapine 3DP ODTs are presented in Figure 4A. All three samples, including the clozapine raw material, 3D-printed clozapine oral disintegrating tablets, and the physical mixture of clozapine and excipients, exhibited characteristic absorption peaks near 188 °C, confirming that this distinct peak belonged to clozapine. Moreover, the number and size of absorption peaks in the curves of the 3D-printed clozapine oral disintegrating tablets and the physical mixture were consistent, indicating that no crystal transformation of clozapine occurred during the 3D printing process. This analysis suggested that there was no interaction between the drug and the excipients. Figure 4B showed the X-ray powder diffractograms of clozapine, the physical mixture, and the clozapine 3DP ODTs. Clozapine displayed multiple sharp Bragg peaks in its XRD pattern, consistent with its crystalline nature. The 3DP ODT data also exhibited the same Bragg peaks, verifying that the drug remained in the crystalline form within the tablets. FTIR spectral interpretation, illustrated in Figure 4C, demonstrated that the characteristic peak of clozapine remained intact, indicating that the molecular structure of clozapine was unchanged. Overall, no evidence of incompatibility between the raw materials and excipients was detected through these analyses.

### 3.4. Characteristic Difference Between 3DP ODTs and Directed Tablets

#### 3.4.1. Disintegration Speed

**Figure 5:**
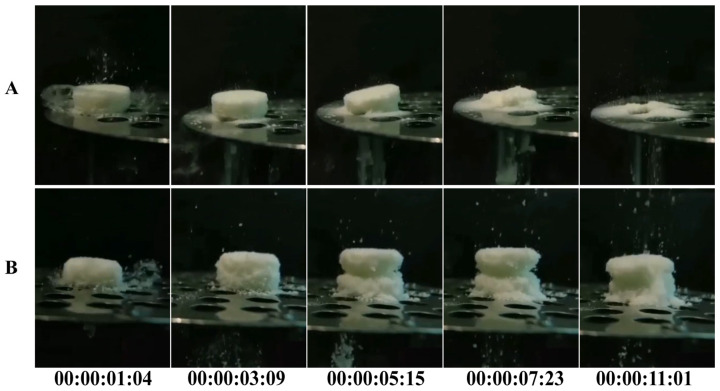
Disintegration process of ODTs: (**A**) 3DP ODTs. (**B**) Direct-pressed tablets.

When two tablets of the same size were placed simultaneously into the dissolution media, significant differences in the disintegration speed of the 3DP tablets and the direct-pressed tablets were observed. As shown in Figure 5 and Video S1, the direct-pressed ODT underwent a water absorption expansion process before disintegration, while the 3DP sheet did not exhibit such expansion and directly disintegrated. The direct-pressed ODT started to collapse only after the 3DP sheet had completely disintegrated, highlighting the significant reduction in disintegration time achieved by the 3DP ODTs. This disparity can be attributed to the unique characteristics of the powder 3D printing process, which results in 3DP ODTs having a low density and a loose, porous structure. Unlike conventional tablets that rely on disintegrants for water absorption and swelling, 3D-printed tablets achieve rapid disintegration through capillary effects generated by their porous structure [36].

#### 3.4.2. Disintegration and Dissolution Tests

**Figure 6:**
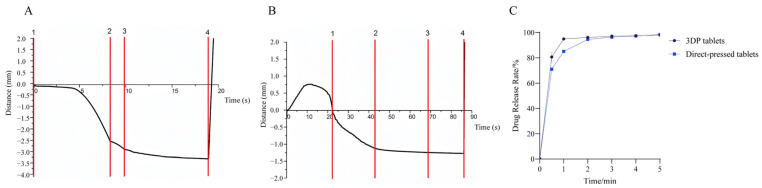
(**A**) Disintegrating curves of 3DP ODTs. (**B**) Disintegrating curves of direct-pressed ODTs. (**C**) Dissolution curve of 3DP ODT and direct-pressed ODT.

Commercial tablets often contain disintegrators such as sodium bicarbonate and citric acid. Before the disintegration of commercial tablets, the disintegrator within the tablet reacts with water to produce gas, causing pre-expansion, disrupting the dense particle structure of the tablet, and creating channels for the dispersion medium to enter the tablet interior [37]. This process enhances the wettability of the tablet, thereby facilitating its disintegration. In contrast, for 3DP ODTs, most of the excipients in the tablet formulation are water-soluble. Hence, these tablets can directly disintegrate upon contact with the dissolving medium water [38]. Additionally, their porous internal structure generated a strong capillary force, enabling the tablets to absorb a large amount of dissolved medium water in a shorter time and achieve complete disintegration [39]. Using Exponent (Version 6.2) software for calculation, as shown in Figure 6A,B, the disintegration time of conventional tablets was 42.9 ± 0.3 s, significantly longer than the 9.9 ± 0.7 s required for 3DP tablets. This marked disparity not only reflects fundamental differences in disintegration mechanisms between the two tablet types but also highlights the superior performance of 3D-printed formulations. Furthermore, the duration of the insoluble dispersion phase between Anchor 2 and Anchor 3 could serve as an indicator for simulating the oral retention time of such materials. Preliminary findings demonstrate that 3D-printed tablets exhibit a notably shorter insoluble dispersion phase compared to direct-pressed tablets. This indicates that 3D-printed tablets achieve faster dissolution upon swallowing, substantially reducing oral foreign body sensation, thereby making them particularly suitable for patients with psychiatric disorders who may experience difficulties in swallowing direct-pressed tablets [40]. Figure 6C revealed that 3DP ODTs achieved 80% drug dissolution within 30 s, outperforming direct-pressed ODTs that only reached 71% dissolution during the same period. At the 1 min mark, 3DP ODTs demonstrated 96% dissolution efficiency, compared to 85% for direct-pressed ODTs. The enhanced dissolution efficiency of 3DP tablets can be attributed to their porous architecture facilitating effective water penetration and the incorporation of higher proportions of water-soluble excipients. These structural advantages enable direct and rapid drug release upon aqueous contact. In contrast, commercial tablets rely on disintegrants that function through water absorption and swelling—a process essential for disrupting their densely packed internal structure to achieve disintegration and drug release. However, this mechanism inherently prolongs the complete dissolution time compared to 3D-printed formulations.

#### 3.4.3. Appearance and Contact Angle

**Figure 7:**
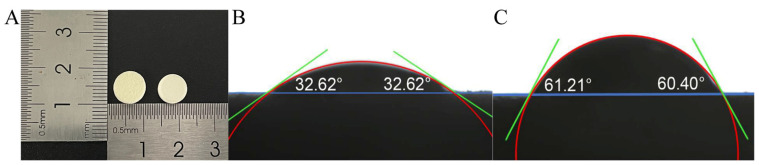
(**A**) Appearance of 3DP ODT (**left**) and direct-pressed ODT (**right**). (**B**) Contact angle of 3DP ODT. (**C**) Contact angle of direct-pressed ODT.

Figure 7A shows that the appearance of the 3D-printed ODTs is comparable to that of the direct-pressed ODTs. The radius of the 3D-printed tablets is 5 mm, with a layer height set at 0.05 mm. This specific layer height was selected to optimize the printing process and to ensure the required physical properties of the tablets, particularly their porosity and disintegration behavior [21]. Figure 7B,C present the contact angle testing results, revealing contact angles between water and the surface of 3DP ODTs and direct-pressed ODTs as 32.82 ± 0.28° and 60.48 ± 0.36°, respectively. This indicated that the 3DP ODTs possess better wettability [27]. Their porous architecture, in conjunction with hydrophilic excipients such as mannitol, facilitates rapid water absorption. The open pore network enables swift liquid permeation throughout the tablet. These factors collectively ensure that the tablet begins to disintegrate almost immediately upon contact with water, without the necessity for excipient swelling or chemical reactions. This phenomenon further emphasizes the strong water absorption and extremely low expansibility of 3DP ODTs.

#### 3.4.4. SEM

**Figure 8:**
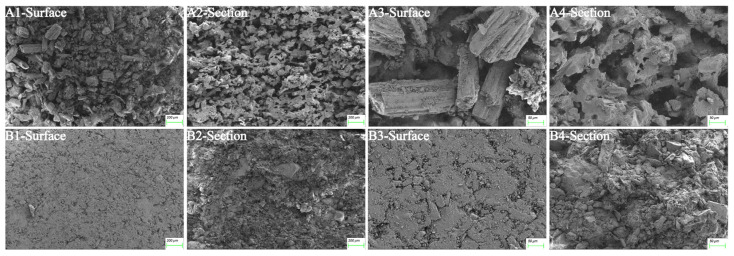
Microscopic morphology of ODTs under SEM across different fields of view: (**A1**–**A4**) 3DP ODTs. (**B1**–**B4**) Direct-pressed tablets.

SEM was used to study the micromorphology of 3DP ODTs (Figure 8(A1)) and direct-pressed ODTs (Figure 8(B1)). The loose porous structure of 3DP ODTs was observed, which is the reason for their high water absorption and easy disintegration. Further examination of the cross-section of the tablet revealed that the pores of the 3DP ODTs exhibited a honeycomb-like structure (Figure 8(A2)), while the direct-pressed ODTs were so closely packed that the pore size was almost invisible (Figure 8(B2)). At a magnification of 50 μm, the microstructural matrix of 3DP ODTs (Figure 8(A3,A4)) had many capillary channels and some large prismatic white crystal particles of excipients, while the particles inside the direct-pressed ODTs connected tightly with few pores and some crushed particles or crystals (Figure 8(B3,B4)). This loose porous structure is the reason for the high water absorption of 3DP ODTs, and it also suggests that the interaction between excipients and excipients is weaker, which is more conducive to disintegration [38]. In contrast, the compact structure of conventional tablets necessitates the use of disintegrants to swell and disrupt interparticulate bonds, thereby delaying disintegration. This comparison highlights the distinct advantage of 3D printing in structural control.

#### 3.4.5. Porosity

The porosity of 3DP clozapine disintegrating tablets was systematically compared to those of tablets produced via direct compression. Traditional tablets typically exhibit small, often closed pores, which impede water penetration and result in a slower disintegration rate. In contrast, the 3DP ODTs possess a loose and porous surface structure that enhances solvent penetration and facilitates rapid tablet disintegration [41]. Furthermore, the presence of additional micropores or void structures in the 3DP ODTs, as compared to direct-pressed tablets, likely contributes to their rapid disintegration without any significant expansion. As shown in Table 2, the total pore volume of the 3DP clozapine ODT was found to be 4.16 times greater that of the direct-pressed tablet, with an average pore size 150 times larger. The porosity of 3D-printed tablets was 48.97%, whereas that of conventionally compressed tablets was a mere 19.06%. This disparity accounts for the more rapid disintegration rate observed in 3D-printed tablets. Moreover, the permeability of the 3DP tablet exceeded that of the direct-pressed tablet by over 4000 times. It is well-established that lower porosity in solid tablets hinders water infiltration and disintegration [42], thus elucidating the superior performance of the 3DP oral disintegrating tablets.

**Figure 9:**
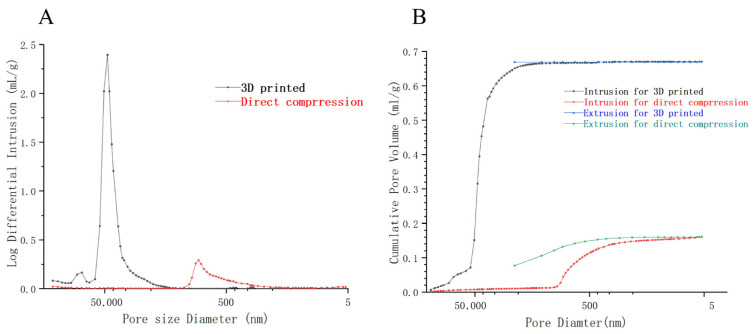
Results of mercury injection method. (**A**) Showing the distribution of pore volume corresponding to the pore size of 3DP and direct-pressed tablets. (**B**) The comparison of mercury pouring and mercury retreating process between the 3DP tablet and the direct-pressed tablet.

Figure 9A shows that when the pore size of 3DP tablets is 20 μm to 70 μm, the pore size at this time is usually distributed, and when the pore size is 45 μm, the pore size reaches the maximum, indicating that the 3DP tablets have large pore size and a large number of pores. In contrast, direct-pressed tablets had a small number of pores and a small size of about 1 μm. The larger the pore of the tablet, the easier it is for a liquid to seep into the inside, and the more the ODTs disintegrate. This phenomenon explains why the 3D-printed oral disintegrating tablets disintegrate faster than the direct-pressed tablets sold on the market. Figure 9B depicts the mercury entry and withdrawal processes of ODTs prepared by two different technologies. It further illustrates that the pores of ODTs prepared by 3D printing are larger than those of direct-pressed tablets, and the number of pores is larger. The mercury removal curves were above the mercury invasion because much energy was absorbed in the mercury process [43]. The mercury retraction curve lies above the intrusion curve due to elastic recovery of the porous material during depressurization, which leads to increased mercury entrapment. The high porosity inside 3DP OTDs was further explained, which provided a robust capillary force and helped the tablet absorb a large amount of dissolved media in a shorter time to achieve complete disintegration [36].

## 4. Conclusions

This study demonstrates the successful integration of CCD and powder-based 3D printing to fabricate disintegrant-free ODTs with tailored performance for patients with dysphagia or urgent therapeutic needs. The optimized formulation achieved rapid disintegration (<10 s) and mechanical robustness (hardness: 35.88 ± 0.43 N) through a unique porous architecture (48.97% porosity) and enhanced hydrophilicity (contact angle: 32.82 ± 0.28°), eliminating reliance on traditional disintegrants. In contrast to conventional compressed tablets, which depend on slow water absorption and swelling mechanisms (disintegration time: 40 s), the 3D-printed ODTs leverage capillary action and open-pore networks for instantaneous drug release, addressing critical limitations in patient compliance and storage stability. By bridging additive manufacturing with pharmaceutical formulation science, this work establishes a scalable framework for patient-centric therapies. The CCD-guided optimization not only balances mechanical and disintegration properties but also provides a paradigm for personalized dosing through structural customization. Future efforts will extend this platform to other poorly soluble drugs and evaluate clinical applicability under real-world conditions, further advancing the role of 3D printing in precision medicine.

## Figures and Tables

**Table 1:** Prescription components of 3D powder-based clozapine ODTs.

Prescription Components	Proportion (*w*/*w*, %)
Clozapine	20%
Mannitol and sucrose	70%
PVP K30 and MCC	10%

**Table 2:** Porosity and other data.

Type	Total Intrusion Volume [10^3^ mL/mg]	Median Pore Diameter V (nm)	Median Pore Diameter A (nm)	Average Pore Diameter (4 V/A)	Porosity (%)	Permeabilitiy (%)
3D printed ODT	0.67	44,560.80	11,615.44	20,992.19	48.97	4606.38
Direct pressing ODT	0.16	971.14	10.25	138.91	19.06	0.96

## Data Availability

Data are contained within the article.

## Supplementary Materials

The following supporting information can be downloaded at https://www.mdpi.com/article/10.3390/pharmaceutics17040435/s1, Video S1. Disintegration process of ODTs.
